# Supplementary material for: Descriptive Epidemiology of Hospitalization of Patients with a Rare Tumor in an Italian Region
Source: Curr Oncol. 2022 Dec 8;29(12):9711–21. doi: 10.3390/curroncol29120762 (PMC9776515; doi:10.3390/curroncol29120762)
Supplement: Supplementary file 1 [file curroncol-29-00762-s001.zip › curroncol-2009375-supplementary.pdf]

Supplementary Tables and Figures

for article

Descriptive epidemiology of hospitalization of patients with a rare tumor in an Italian Region

Rosa Alessandra[1], Fontana Vincenzo[1], Filiberti Rosa Angela[1], Pronzato Paolo[2], Mannucci Matilde[1].

1 - IRCCS Ospedale Policlinico San Martino, Clinical Epidemiology Unit, Genoa, Italy

2 - IRCCS Ospedale Policlinico San Martino, Medical Oncology Unit, Genoa, Italy

Corresponding author:

Rosa Alessandra, IRCCS Ospedale Policlinico San Martino, Clinical Epidemiology, L.go Rosana Benzi, 10 16132 Genoa, Italy; email: alessandra.rosa@hsanmartino.it.

Supplementary Table S1. Selection of patients with rare tumors according to ICD-9-CM classification in discharge diagnosis.

Code	Tumor Groups	Site	ICD-9-CM*
SAR	Sarcomas	Bone and articular cartilage, connective and soft tissue	170-171
CNS	Central nervous system	Eye, brain, other and unspecified parts of nervous system	190-192
HNT	Epithelial T of head and neck	Tongue, major salivary glands, gums, mouth, oro-/naso-pharynx	141-147, 149
HET	Hematological malignancies	Multiple myeloma and immunoproliferative neoplasms, leukemias	203-208
TCT	Epithelial T of thoracic cavity	Trachea, pleural mesothelioma, thymus	162.1, 163, 164.0
GUT	Urinary system and male genital organs	Testis, penis, other male genital organs; renal pelvis, ureter, urethra	186-187, 189.1, 189.2, 189.3
FGT	Female genital system	Vagina, clitoris and vulva	184.0, 184.3, 184.4
DGT	Epithelial T of digestive system	Small intestine, anus, liver, gallbladder, bile ducts	152, 154.3, 155-156
EGT	T of endocrine glands	Including thyroid	193-194
SKT	Epithelial T of skin	Skin of trunk except scrotum; labia	173.5, 184.1, 184.2

* International Classification of Disease, Nine Revision, Clinical Modification

Supplementary Table S2. Distribution of age at H_0 by group of rare tumors.

Tumor Groups	Age at H_0 [a]				
	0-20		21-103		Total
	N[§]	%[*]	N[§]	%[*]	N[§]
Sarcomas (SAR)	331	11.29	2601	88.71	2932
Central nervous system (CNS)	889	11.41	6900	88.59	7789
Head and neck (HNT)	21	0.47	4461	99.53	4482
Hematological (HET)	692	5.43	12055	94.57	12747
Thoracic cavity (TCT)	0	0.00	3458	100.00	3458
Genitourinary system (GUT)	49	1.32	3664	98.68	3713
Female genital system (FGT)	0	0.00	967	100.00	967
Digestive system (DGT)	23	0.20	11687	99.80	11710
Endocrine glands (EGT)	422	8.05	4820	91.95	5242
Skin (SKT)	9	0.21	4280	99.79	4289
Total	2436	4.25	54893	95.75	57329

[a] index hospitalization (first admission); [§] total admission; [*] calculated on the total at the previous admission

Supplementary Table S3. Distribution of re-hospitalization by group of rare tumors and calendar period.

Sarcomas (SAR)

Period		Re-hospitalization													
	H_0 [a]	First		Second		Third		Fourth		Fifth		Tenth		Fifteenth	
	N[§]	N[§]	%[*]	N[§]	%[*]	N[§]	%[*]	N[§]	%*	N[§]	%[*]	N[§]	%[*]	N[§]	%[*]
2000-2004	972	520	53	344	66	275	80	219	80	184	84	97	88	54	87
2005-2009	709	366	52	243	66	182	75	153	84	131	86	71	90	39	93
2010-2014	625	291	47	196	67	159	81	121	76	96	79	50	93	31	97
2015-2019	626	280	45	180	64	138	77	112	81	87	78	48	84	19	79
Total	2932	1457	50	963	66	754	78	605	80	498	82	266	89	143	89

Central Nervous System (CNS)

Period		Re-hospitalization													
	H_0 [a]	First		Second		Third		Fourth		Fifth		Tenth		Fifteenth	
	N[§]	N[§]	%[*]	N[§]	%[*]	N[§]	%[*]	N[§]	%*	N[§]	%[*]	N[§]	%[*]	N[§]	%[*]
2000-2004	2111	1142	54	740	65	517	70	383	74	311	81	134	86	75	88
2005-2009	1932	991	51	627	63	433	69	329	76	253	77	91	83	57	86
2010-2014	1949	949	49	610	64	437	72	302	69	223	74	90	88	49	84
2015-2019	1797	852	47	456	54	269	59	168	62	109	65	34	77	14	100
Total	7789	3934	51	2433	62	1656	68	1182	71	896	76	349	85	195	87

Head and neck (HNT)

Period		Re-hospitalization													
	H_0 [a]	First		Second		Third		Fourth		Fifth		Tenth		Fifteenth	
	N[§]	N[§]	%[*]	N[§]	%[*]	N[§]	%[*]	N[§]	%*	N[§]	%[*]	N[§]	%[*]	N[§]	%[*]
2000-2004	1519	977	64	635	65	442	70	312	71	207	66	40	75	3	50
2005-2009	1169	783	67	505	64	338	67	237	70	145	61	17	55	4	100
2010-2014	941	586	62	334	57	198	59	116	59	68	59	8	100	2	67
2015-2019	853	401	47	196	49	116	59	60	52	35	58	3	75	1	100
Total	4482	2747	61	1670	61	1094	66	725	66	455	63	68	71	10	71

Supplementary Table S3. (continued)

Hematological (HET)

Period	H_0 [a]	Re-hospitalization													
		First		Second		Third		Fourth		Fifth		Tenth		Fifteenth	
	N[§]	N[§]	%[*]	N[§]	%[*]	N[§]	%[*]	N[§]	%*	N[§]	%[*]	N[§]	%[*]	N[§]	%[*]
2000-2004	4391	3341	76	2644	79	2137	81	1751	82	1467	84	691	88	341	87
2005-2009	3407	2418	71	1900	79	1558	82	1297	83	1094	84	527	88	226	82
2010-2014	2664	1986	75	1532	77	1242	81	1009	81	841	83	331	84	121	79
2015-2019	2280	1534	67	1136	74	857	75	689	80	520	75	171	81	70	80
Total	12742	9279	73	7212	78	5794	80	4746	82	3922	83	1720	86	758	83

Thoracic cavity (TCT)

Period	H_0	Re-hospitalization													
		First		Second		Third		Fourth		Fifth		Tenth		Fifteenth	
	N[§]	N[§]	%[*]	N[§]	%[*]	N[§]	%[*]	N[§]	%*	N[§]	%[*]	N[§]	%[*]	N[§]	%[*]
2000-2004	973	666	68	489	73	353	72	276	78	212	77	42	75	8	62
2005-2009	930	658	71	487	74	354	73	247	70	170	69	27	75	6	75
2010-2014	852	581	68	412	71	287	70	195	68	126	65	7	35	1	100
2015-2019	702	441	63	257	58	146	57	89	61	41	46	2	100	0	0
Total	3457	2346	68	1645	70	1140	69	807	71	549	68	78	68	15	68

Genitourinary system (GUT)

Period	H_0 [a]	Re-hospitalization													
		First		Second		Third		Fourth		Fifth		Tenth		Fifteenth	
	N[§]	N[§]	%[*]	N[§]	%[*]	N[§]	%[*]	N[§]	%*	N[§]	%[*]	N[§]	%[*]	N[§]	%[*]
2000-2004	920	438	48	217	50	110	51	73	66	52	71	12	75	3	75
2005-2009	897	382	43	173	45	85	49	52	61	32	62	6	100	0	0
2010-2014	924	416	45	155	37	72	46	45	63	31	69	5	100	0	0
2015-2019	972	389	40	142	37	72	51	38	53	22	58	2	40	0	0
Total	3713	1625	44	687	42	339	49	208	61	137	66	25	78	3	60

Supplementary Table S3. (continued)

Female genital system (FGT)

Period	H_0 [a]	Re-hospitalization													
		First		Second		Third		Fourth		Fifth		Tenth		Fifteenth	
	N[§]	N[§]	%[*]	N[§]	%[*]	N[§]	%[*]	N[§]	%*	N[§]	%[*]	N[§]	%[*]	N[§]	%[*]
2000-2004	285	164	58	88	54	55	63	38	69	24	63	1	50	1	100
2005-2009	249	136	55	71	52	39	55	23	59	14	61	2	100	0	0
2010-2014	215	112	52	66	59	42	64	20	48	13	65	0	0	0	0
2015-2019	218	81	37	40	49	18	45	11	61	4	36	0	0	0	0
Total	967	493	51	265	54	154	58	92	60	55	60	3	60	1	100

Digestive system (DGT)

Period	H_0 [a]	Re-hospitalization													
		First		Second		Third		Fourth		Fifth		Tenth		Fifteenth	
	N[§]	N[§]	%[*]	N[§]	%[*]	N[§]	%[*]	N[§]	%*	N[§]	%[*]	N[§]	%[*]	N[§]	%[*]
2000-2004	3457	2115	61	1405	66	1006	72	766	76	576	75	163	77	54	76
2005-2009	3016	1869	62	1293	69	935	72	681	73	505	74	149	82	37	76
2010-2014	2671	1615	60	1024	63	702	69	496	71	334	67	48	67	8	53
2015-2019	2560	1318	51	748	57	421	56	255	61	147	58	15	71	4	100
Total	11704	6917	59	4470	65	3064	69	2198	72	1562	71	375	77	103	74

Endocrine glands (EGT)

Period	H_0 [a]	Re-hospitalization													
		First		Second		Third		Fourth		Fifth		Tenth		Fifteenth	
	N[§]	N[§]	%[*]	N[§]	%[*]	N[§]	%[*]	N[§]	%*	N[§]	%[*]	N[§]	%[*]	N[§]	%[*]
2000-2004	1534	943	61	561	59	339	60	240	71	186	78	58	78	15	79
2005-2009	1333	687	52	361	53	214	59	137	64	102	74	26	76	10	91
2010-2014	1209	481	40	218	45	116	53	83	72	63	76	19	68	7	78
2015-2019	1166	296	25	107	36	59	55	42	71	31	74	5	56	1	100
Total	5242	2407	46	1247	52	728	58	502	69	382	76	108	74	33	83

Supplementary Table S3. (continued)

Skin (SKT)

Period	H_0[a]	Re-hospitalization													
		First		Second		Third		Fourth		Fifth		Tenth		Fifteenth	
	N[§]	N[§]	%[*]	N[§]	%[*]	N[§]	%[*]	N[§]	%*	N[§]	%[*]	N[§]	%[*]	N[§]	%[*]
2000-2004	1274	289	23	114	39	58	51	36	62	25	69	8	89	1	50
2005-2009	1276	239	19	87	36	42	48	29	69	17	59	4	80	1	100
2010-2014	865	126	15	41	33	23	56	10	43	7	70	0	0	0	0
2015-2019	873	93	11	30	32	15	50	6	40	3	50	0	0	0	0
Total	4288	747	17	272	36	138	51	81	59	52	64	12	86	2	67

[a] index hospitalization (first admission); [§] total admission; [*] calculated on the total at the previous admission

Supplementary Figure S1. Median timespan and 95% confidence interval between two consecutive hospitalizations for rare tumor in Sarcomas in Liguria Region, 2000-2019

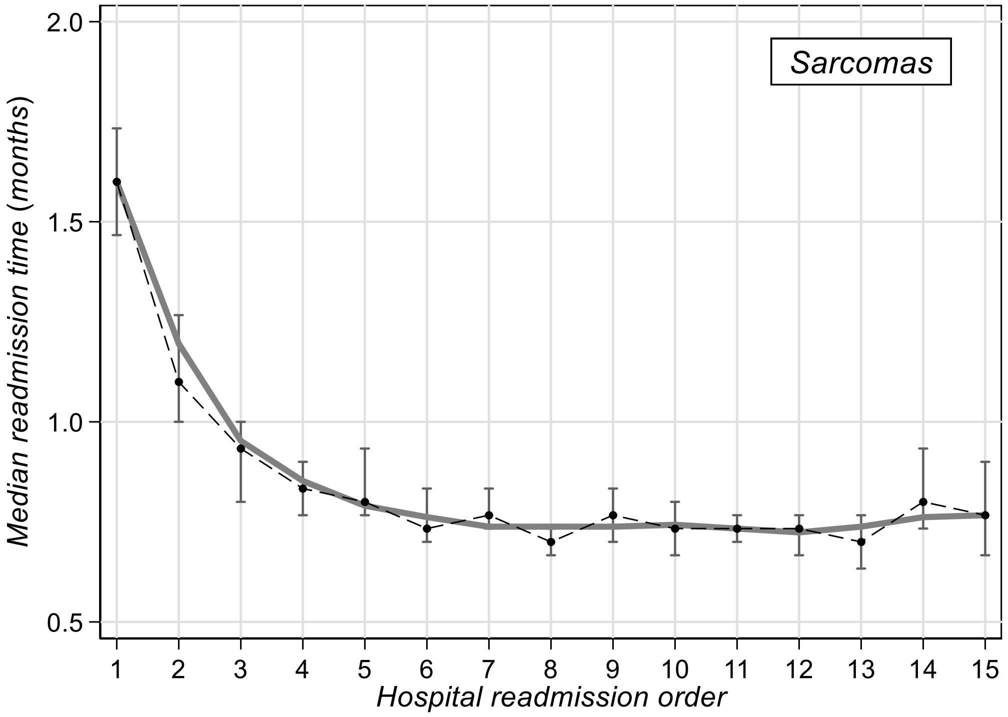

Supplementary Figure S2. Median timespan and 95% confidence interval between two consecutive hospitalizations for rare tumor in central nervous system tumor in Liguria Region, 2000-2019

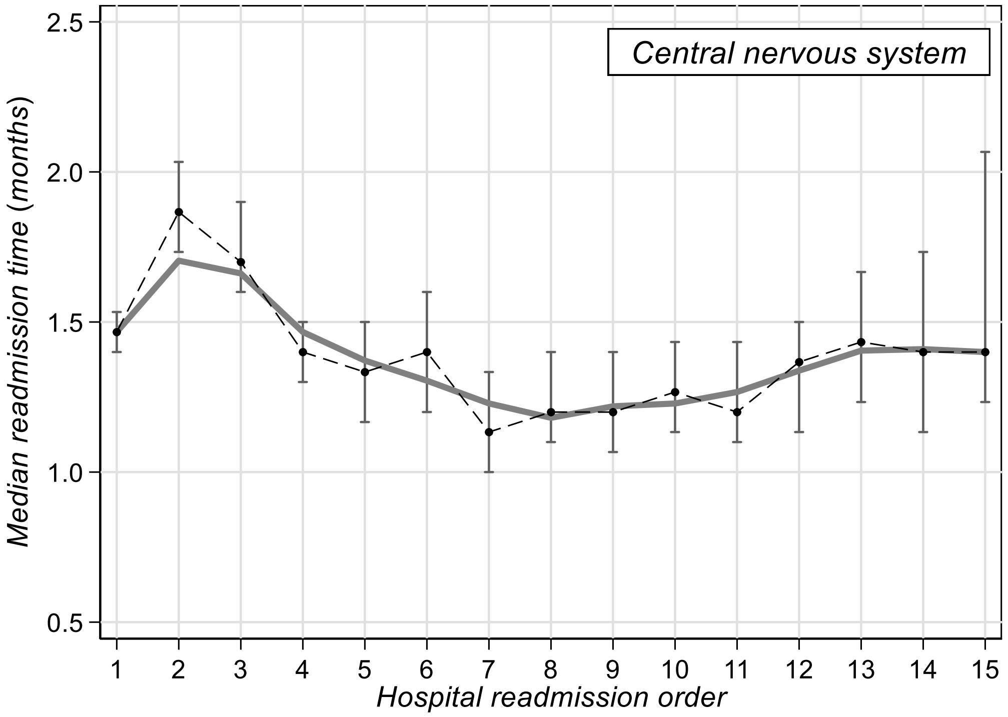

Supplementary Figure S3. Median timespan and 95% confidence interval between two consecutive hospitalizations for rare tumor in Head and neck tumor system in Liguria Region, 2000-2019

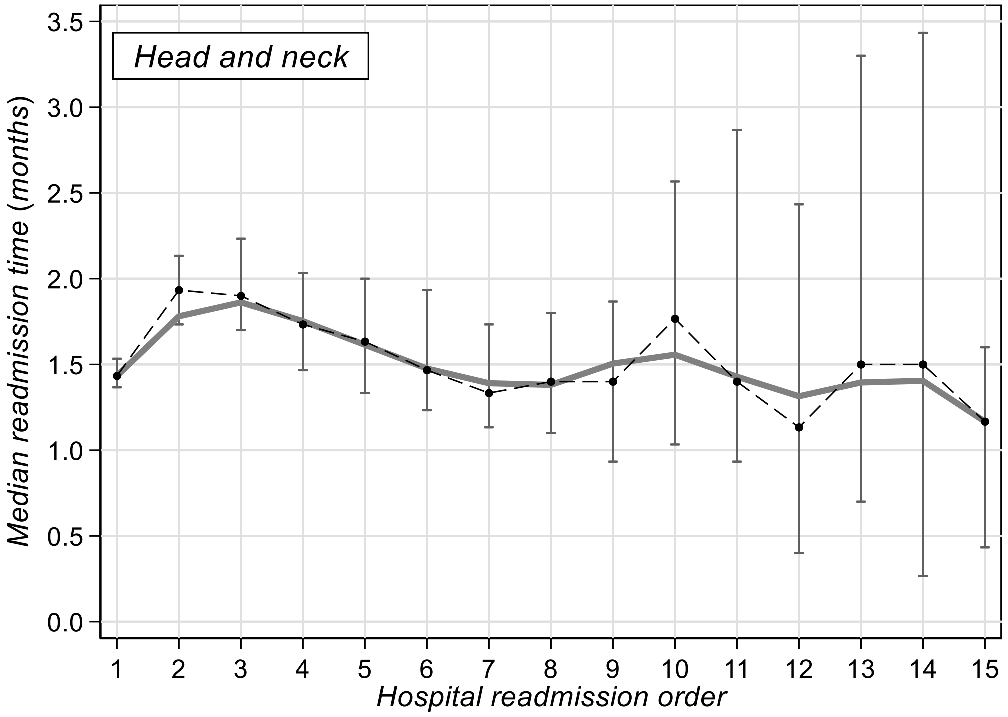

Supplementary Figure S4. Median timespan and 95% confidence interval between two consecutive hospitalizations for rare tumor in hematological tumor in Liguria Region, 2000-2019

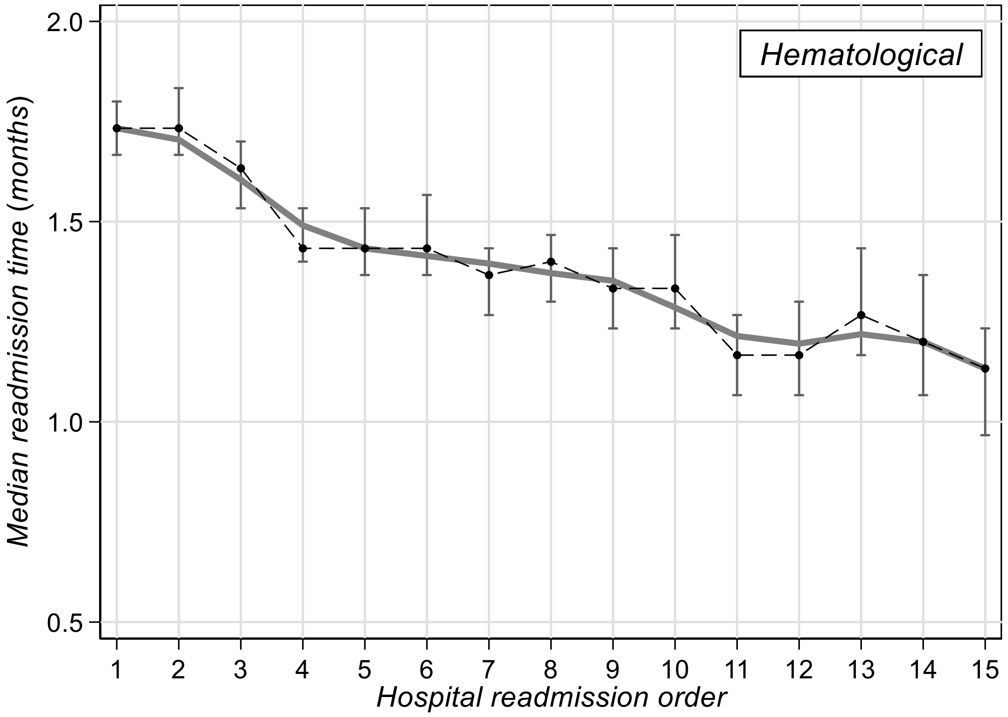

Supplementary Figure S5. Median timespan and 95% confidence interval between two consecutive hospitalizations for rare tumor in thoracic cavity tumor in Liguria Region, 2000-2019

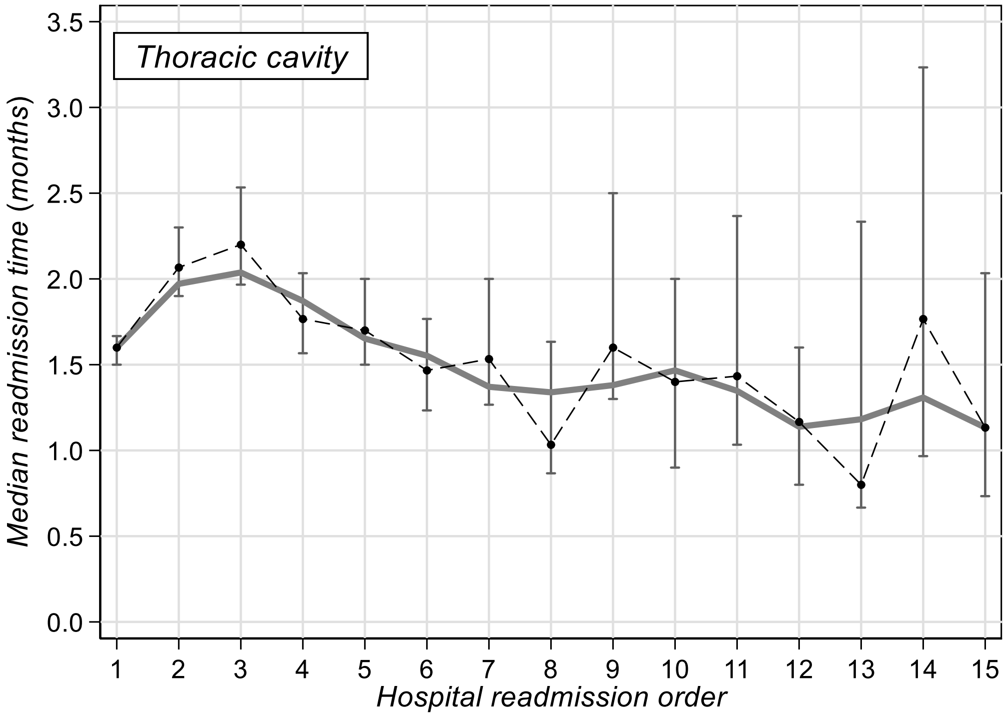

Supplementary Figure S6. Median timespan and 95% confidence interval between two consecutive hospitalizations for rare tumor in genitourinary system tumor in Liguria Region, 2000-2019

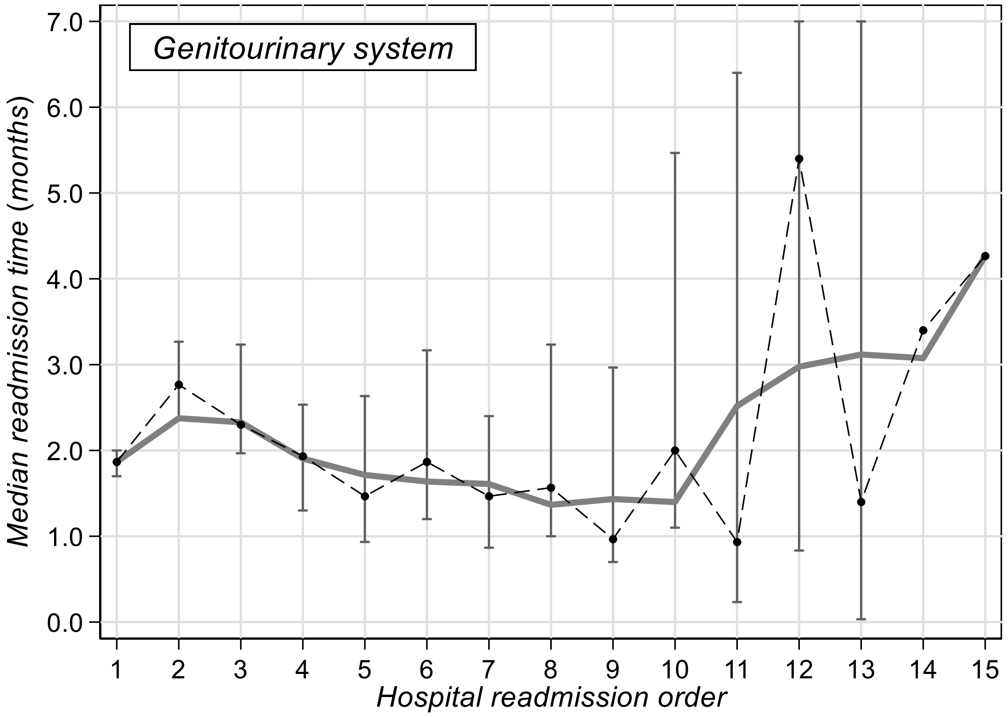

Supplementary Figure S7. Median timespan and 95% confidence interval between two consecutive hospitalizations for rare tumor in female genital system tumor in Liguria Region, 2000-2019

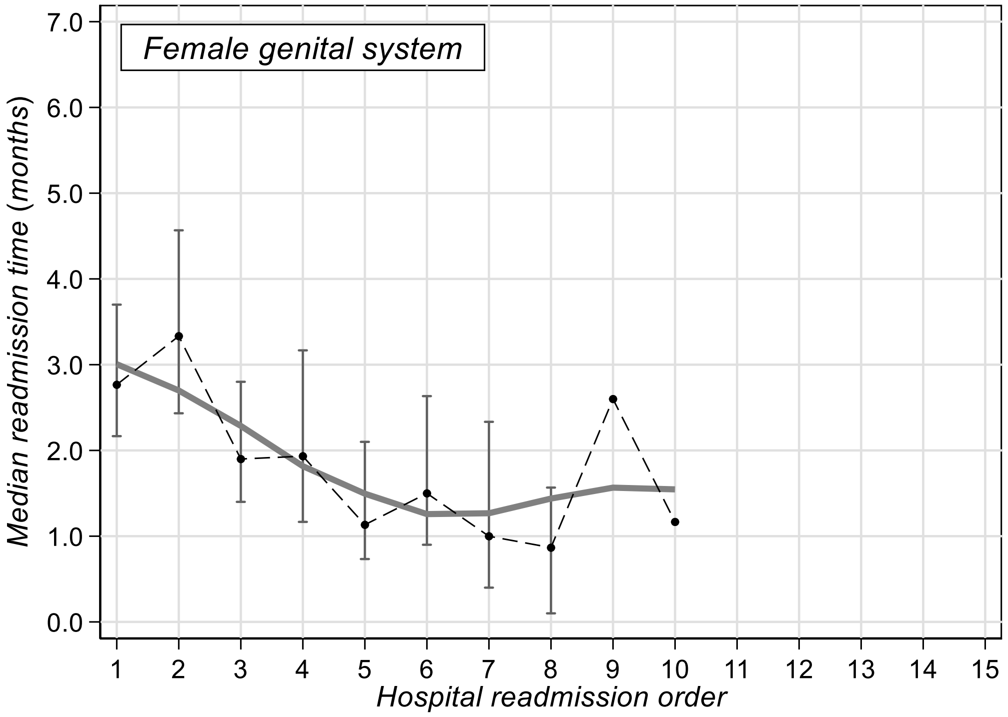

Supplementary Figure S8. Median timespan and 95% confidence interval between two consecutive hospitalizations for rare tumor in digestive system tumor in Liguria Region, 2000-2019

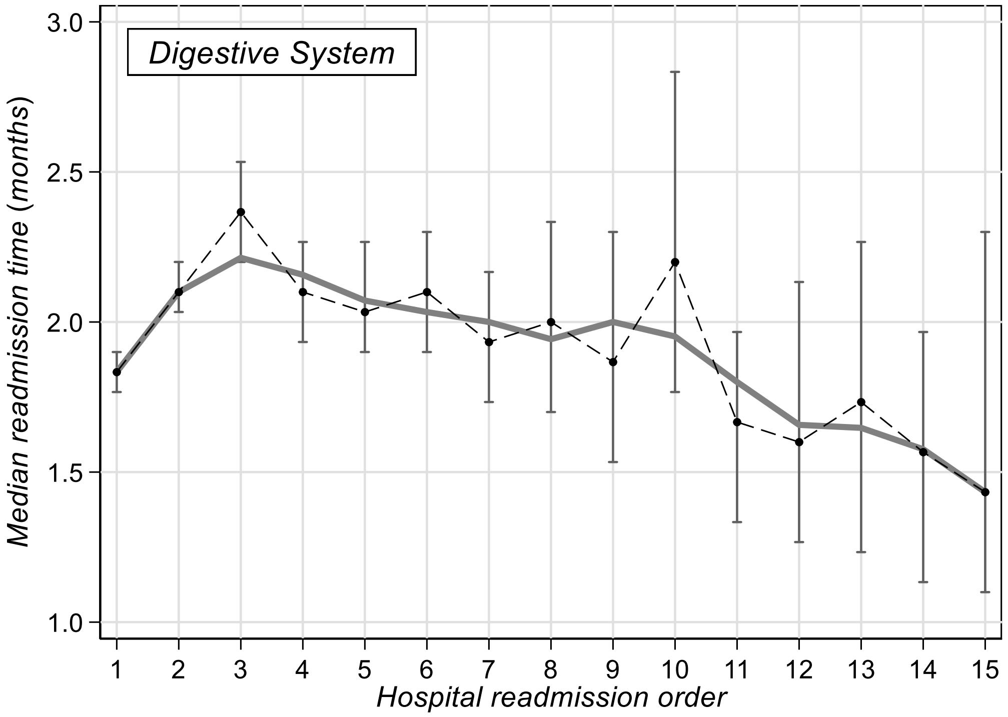

Supplementary Figure S9. Median timespan and 95% confidence interval between two consecutive hospitalizations for rare tumor in endocrine glands tumor in Liguria Region, 2000-2019

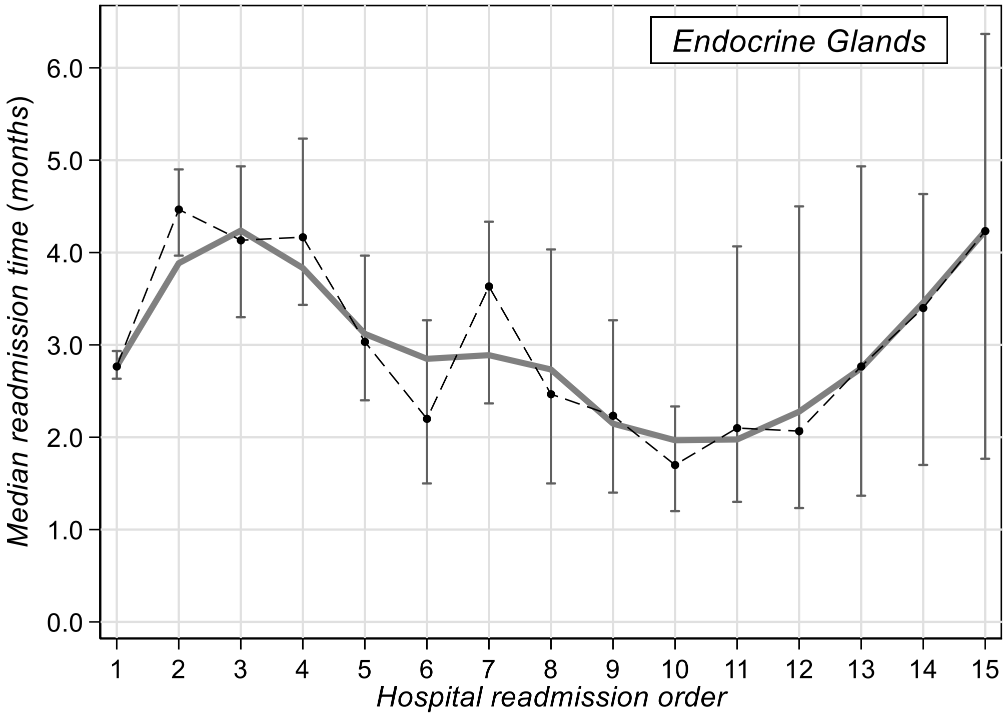

Supplementary Figure S10. Median timespan and 95% confidence interval between two consecutive hospitalizations for rare tumor in skin tumor in Liguria Region, 2000-2019

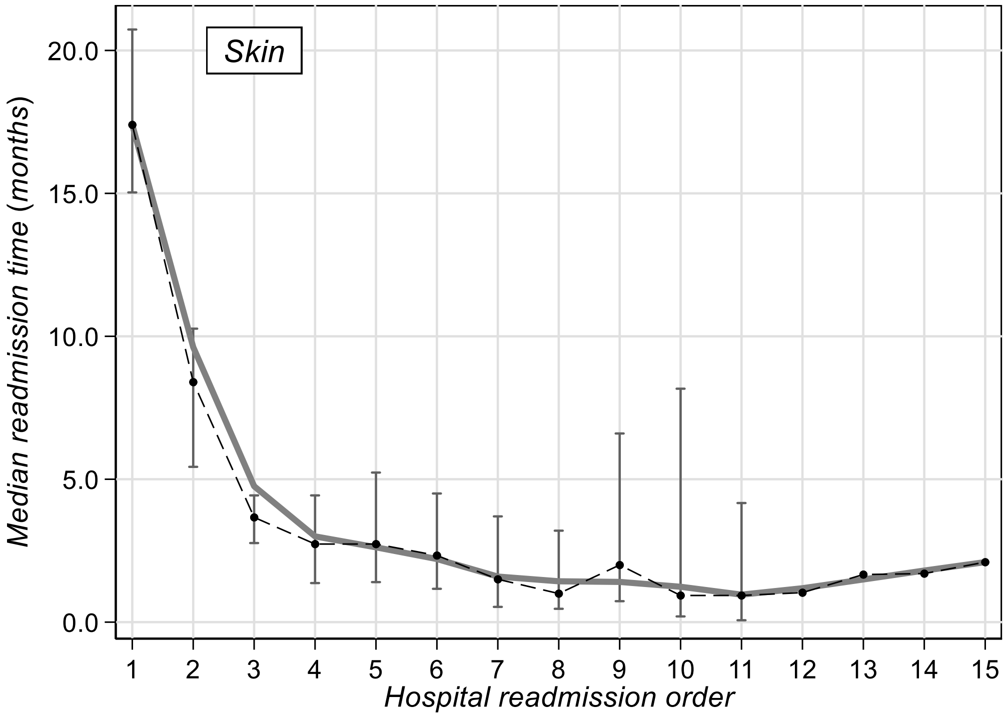

Supplementary Table S4. Type of hospitalization of patients with rare tumors in Liguria Region during 2000-2019.

Type of hospitalization	Hospitalizations at H_0[a]		All hospitalizations	
	N[§]	%[*]	N[§]	%[*]
Planned – No Emergency	19430	33.9	66492	36.2
Emergency	18062	31.5	47185	25.7
Mandatory Medical Treatment	6	0.1	19	0.1
Planned – pre-hospitalization	2205	3.9	3963	2.2
Missing data	17626	30.8	66300	36.0
Total	57329	100.0	183959	100.0

[a] index hospitalization (first admission); [§] absolute frequency; [*] percent frequency.

Supplementary Table S5. Regimen of hospitalization of patients with rare tumors in Liguria Region during 2000-2019.

Regimen of hospitalization	Hospitalizations at H_0[a]		All hospitalizations	
	N[§]	%[*]	N[§]	%[*]
Ordinary	38879	67.8	115316	62.7
Day Hospital	18192	31.7	67817	36.9
Missing data	258	0.4	826	0.5
Total	57329	100.0	183959	100.0

[a] index hospitalization (first admission); [§] absolute frequency; [*] percent frequency

Supplementary Table S6. Hospitalization regimen in patients with rare tumors in Liguria Region during 2000-2019 by calendar period and re-hospitalization order.

Hospitalization	Period	Ordinary		Day Hospital		Missing data		Total
		N[§]	%[*]	N[§]	%[*]	N[§]	%[*]	
H_0 [a]	2000-2004	11342	65	6094	35	0	0	17436
	2005-2009	9659	65	5259	35	0	0	14918
	2010-2014	8596	67	4061	31	258	2	12915
	2015-2019	9274	77	2773	23	0	0	12047
	Total	38871	68	18187	32	258	0	57316
First	2000-2004	6904	65	3687	35	4	0	10595
	2005-2009	5529	65	2997	35	3	0	8529
	2010-2014	4678	65	2303	32	162	2	7143
	2015-2019	3822	67	1863	33	0	0	5685
	Total	20933	66	10850	34	169	1	31952
Second	2000-2004	4450	61	2784	38	3	0	7237
	2005-2009	3483	61	2259	39	5	0	5747
	2010-2014	2777	61	1729	38	82	2	4588
	2015-2019	2115	64	1177	36	0	0	3292
	Total	12825	61	7949	38	90	0	20864
Third	2000-2004	3195	60	2093	40	4	0	5292
	2005-2009	2495	60	1678	40	7	0	4180
	2010-2014	1996	61	1224	37	58	2	3278
	2015-2019	1382	65	729	35	0	0	2111
	Total	9068	61	5724	39	69	0	14861
Fourth	2000-2004	2462	60	1632	40	0	0	4094
	2005-2009	1929	61	1251	39	5	0	3185
	2010-2014	1431	60	925	39	41	2	2397
	2015-2019	962	65	508	35	0	0	1470
	Total	6784	61	4316	39	46	0	11146
Fifth	2000-2004	1919	59	1323	41	2	0	3244
	2005-2009	1401	57	1057	43	5	0	2463
	2010-2014	1062	59	707	39	33	2	1802
	2015-2019	650	65	349	35	0	0	999
	Total	5032	59	3436	40	40	0	8508
Tenth	2000-2004	693	56	553	44	0	0	1246
	2005-2009	528	57	390	42	2	0	920
	2010-2014	296	53	258	46	4	1	558
	2015-2019	187	67	93	33	0	0	280
	Total	1704	57	1294	43	6	0	3004
Fifteenth	2000-2004	286	52	269	48	0	0	555
	2005-2009	185	49	195	51	0	0	380
	2010-2014	113	52	105	48	1	0	219
	2015-2019	68	62	41	38	0	0	109
	Total	652	52	610	48	1	0	1263

[a] index hospitalization (first admission); [§] absolute frequency; [*] percent frequency

Supplementary Table S7. Reasons for hospitalization and re-hospitalization in day hospital regimen of patients with rare tumors in Liguria Region during 2000-2019 by readmission order and calendar period.

Hospitalization	Period	Diagnostic		Surgery Day surgery		Therapeutic		Rehabilitation		Missing data		Total
		N[§]	%[*]	N[§]	%[*]	N[§]	%[*]	N[§]	%[*]	N[§]	%[*]	
H_0 [a]	2000-2004	3311	54	1468	24	615	10	13	0	687	11	6094
	2005-2009	1840	35	1089	21	1631	31	8	0	691	13	5259
	2010-2014	664	16	749	18	1598	39	6	0	1044	26	4061
	2015-2019	291	10	1068	39	1133	41	0	0	281	10	2773
	Total	6106	34	4374	24	4977	27	27	0	2703	15	18187
First	2000-2004	1771	48	640	17	911	25	7	0	358	10	3687
	2005-2009	860	29	216	7	1591	53	9	0	321	11	2997
	2010-2014	288	13	170	7	1622	70	4	0	219	10	2303
	2015-2019	128	7	152	8	1557	84	3	0	23	1	1863
	Total	3047	28	1178	11	5681	52	23	0	921	8	10850
Second	2000-2004	1308	47	380	14	858	31	10	0	228	8	2784
	2005-2009	612	27	95	4	1341	59	9	0	202	9	2259
	2010-2014	199	12	61	4	1370	79	6	0	93	5	1729
	2015-2019	66	6	56	5	1048	89	3	0	4	0	1177
	Total	2185	27	592	7	4617	58	28	0	527	7	7949
Third	2000-2004	972	46	230	11	726	35	4	0	161	8	2093
	2005-2009	501	30	63	4	992	59	11	1	111	7	1678
	2010-2014	165	13	42	3	959	78	3	0	55	4	1224
	2015-2019	48	7	30	4	650	89	1	0	0	0	729
	Total	1686	29	365	6	3327	58	19	0	327	6	5724
Fourth	2000-2004	719	44	168	10	617	38	4	0	124	8	1632
	2005-2009	348	28	52	4	762	61	7	1	82	7	1251
	2010-2014	128	14	18	2	745	81	2	0	32	3	925
	2015-2019	58	11	17	3	432	85	0	0	1	0	508
	Total	1253	29	255	6	2556	59	13	0	239	6	4316
Fifth	2000-2004	609	46	95	7	517	39	4	0	98	7	1323
	2005-2009	328	31	28	3	632	60	6	1	63	6	1057
	2010-2014	115	16	16	2	548	78	2	0	26	4	707
	2015-2019	45	13	8	2	295	85	0	0	1	0	349
	Total	1097	32	147	4	1992	58	12	0	188	5	3436
Tenth	2000-2004	251	45	20	4	240	43	0	0	42	8	553
	2005-2009	133	34	3	1	235	60	0	0	19	5	390
	2010-2014	81	31	6	2	163	63	0	0	8	3	258
	2015-2019	27	29	6	6	60	65	0	0	0	0	93
	Total	492	38	35	3	698	54	0	0	69	5	1294
Fifteenth	2000-2004	134	50	4	1	114	42	0	0	17	6	269
	2005-2009	93	48	2	1	96	49	0	0	4	2	195
	2010-2014	44	42	0	0	58	55	0	0	3	3	105
	2015-2019	18	44	1	2	22	54	0	0	0	0	41
	Total	289	47	7	1	290	48	0	0	24	4	610

[a] index hospitalization (first admission); [§] absolute frequency; [*] percent frequency

Supplementary Table S8. Joint effect of tumor group, gender, age at H0, period of H0 and vital status at last discharge on re-hospitalization rates of patients with rare tumors in Liguria Region during 2000-2019, estimated through the multivariable negative-binomial regression method.

Characteristics & categories	Rate[c]	95%CI[e]	RR[d]	95%CI[e]	P-value[f]
Tumor Groups					<0.001
Sarcomas (SAR)	6.68	5.94-7.51	1.00	(Ref.)[g]	
Central nervous system (CNS)	7.40	6.61-8.29	1.11	0.99-1.25	
Head and neck (HNT)	5.57	4.96-6.26	0.83	0.74-0.94	
Hematological (HET)	6.44	5.76-7.21	0.97	0.86-1.08	
Thoracic cavity (TCT)	7.58	6.72-8.55	1.14	1.01-1.28	
Genitourinary system (GUT)	5.59	4.94-6.33	0.84	0.74-0.95	
Female genital system (FGT)	4.64	4.00-5.39	0.70	0.59-0.81	
Digestive system (DGT)	6.80	6.07-7.62	1.02	0.91-1.14	
Endocrine glands (EGT)	4.90	4.34-5.52	0.73	0.65-0.83	
Skin (SKT)	2.17	1.90-2.47	0.32	0.29-0.37	
Gender					0.106
Male	2.27	2.00-2.58	1.00	(Ref.)[g]	
Female	2.17	1.90-2.47	0.95	0.90-1.01	
Age at H_0[a]					<0.001
0-56	2.13	1.87-2.43	1.00	(Ref.)[g]	
57-69	1.65	1.45-1.88	0.77	0.72-0.84	
70-78	1.70	1.50-1.93	0.80	0.74-0.86	
79-103	2.17	1.90-2.47	1.02	0.94-1.10	
Period of H_0[a]					<0.001
2000-2004	0.92	0.81-1.04	1.00	(Ref.)[g]	
2005-2009	1.01	0.89-1.15	1.10	1.02-1.19	
2010-2014	1.15	1.01-1.30	1.25	1.16-1.35	
2015-2019	2.17	1.90-2.47	2.35	2.18-2.55	
Vital status[b]					<0.001
Alive	1.79	1.57-2.03	1.00	(Ref.)[g]	
Died	2.17	1.90-2.47	1.21	1.15-1.28	
Constant[h]	3.10	2.85-3.35	-	-	-

[a] index hospitalization (first admission); [b] vital status at last discharge; [c] re-hospitalization rate per patient/year; [d] rate ratio; [e] 95% confidence interval for Rate/RR; [f] probability level of the likelihood ratio test; [g] reference category; [h] overall mean rate.
Note: rates and 95%CI were estimated through the negative-binomial distribution.
